# *Coxiella burnetii* Infection Among Blood Donors From Baden‐Wuerttemberg Province and Hesse Province, Germany: A Multicentre Cross‐Sectional Serological Study

**DOI:** 10.1002/puh2.70284

**Published:** 2026-06-16

**Authors:** Larissa Dangel, Daniela Hummel, Volkhard A. J. Kempf, Mirko Faber, Kai Hourfar, Brockmann Stefan, Martin Eichner, Silke F. Fischer

**Affiliations:** ^1^ State Health Office, Ministry of Social Affairs Health and Integration Baden‐Wuerttemberg Stuttgart Germany; ^2^ ECDC Fellowship Programme Public Health Microbiology Path (EUPHEM), European Centre for Disease Prevention and Control (ECDC) Stockholm Sweden; ^3^ Q Fever Consulting Laboratory Baden‐Württemberg State Health Office Stuttgart Germany; ^4^ Institute of Medical Microbiology and Infection Control Goethe University Frankfurt/Main Germany; ^5^ Department for Infectious Disease Epidemiology Robert Koch Institute Berlin Germany; ^6^ German Red Cross, Baden‐Wuerttemberg‐Hesse Institute of Transfusion Medicine and Immuno‐Hemotherapy Goethe University Frankfurt/Main Germany; ^7^ Institute for Clinical Epidemiology and Applied Biometrics University of Tuebingen Tuebingen Germany

**Keywords:** blood donors, *Coxiella burnetii*, Q fever, seroprevalence

## Abstract

In recent years, several large outbreaks have shown that Q fever is a serious disease that unfortunately is underreported in Europe. Furthermore, no seroprevalence data for *Coxiella burnetii* in the general German population are available. The aim of this study is to examine the seroprevalence of *C. burnetii* in a normal, non‐risk population in Germany. To this end, blood donors in the federal states Baden‐Wuerttemberg and Hesse were screened for *C. burnetii‐*specific antibodies as well as associated risk factors. Of over 5000 participants without prior knowledge of a *C. burnetii* infection, 153 (2.96%) were seropositive for anti‐*C. burnetii* antibodies. Place of residence and contact with animals represent increased risk factors, as do occupations closely related to animals. Furthermore, it was shown that in high‐risk occupations, prevalence is higher in younger age groups than in older ones.

## Graphical abstract

This study analyzes the seroprevalence of Coxiella burnetii in a German non‐risk population using a stratified sampling approach across two federal states (n = 8400 potential samples). Screening of over 5000 blood donors reveals an overall seroprevalence of 2.96%. Key risk factors include regional residence, animal contact, and specific high‐risk occupations.

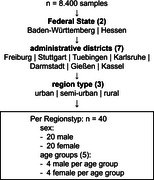

## 1 Introduction

The intracellular, Gram‐negative and highly pathogenic bacterium *Coxiella burnetii* is the causative agent of Q fever (QF), a worldwide zoonosis and was first described in 1937 by Derrick in Brisbane, Australia [1, 2]. Zoonotic infections caused by bacteria, viruses or parasites have become more important in the last years. In Europe, major QF outbreaks have occurred with high numbers of infected people. The largest ever recorded outbreak took place in the Netherlands from 2007 to 2010 with over 4000 notified cases [3]. In Germany, several large outbreaks have also been registered [4, 5]. Nevertheless, knowledge about the occurrence and prevalence of QF remains limited. Therefore, it can be assumed that the number of unreported cases is high.

A first serological survey of different zoonotic pathogens was conducted in Germany in 2015 with over 700 forest workers, a population group at higher risk of exposure to zoonotic pathogens. In this study, the seroprevalence of *C. burnetii* was 6.5% [6]. Studies have investigated the occurrence of QF in high‐risk populations such as veterinarians, shepherds and abattoir workers in Australia and the Netherlands [7, 8], but there is a lack of knowledge regarding the prevalence of QF in the general population in Germany. This is due to the fact that most infections are asymptomatic and thus go unnoticed. Even when symptoms occur, they are often non‐specific and resemble a mild flu‐like illness, which can include fever, headache, myalgia, and respiratory symptoms [9, 10]. Approximately 1%–5% of patients with acute QF become chronic [11]. Chronic QF can manifest as endocarditis, granulomatous hepatitis or vascular infections, conditions associated with high mortality ranging from 20% to 40%, increasing to up to 60% if left untreated [12, 13, 14]. Serological testing allows differentiation between the acute and chronic phases; the indirect immunofluorescence assay (IFA) remains the gold standard for quantifying antibody titres across immunoglobulin classes and confirming chronic infection. In the early stages of acute QF or during chronic phase, PCR is also a valuable diagnostic tool [15].

As widely reported in the literature, humans and animals can acquire *C. burnetii* infection through several transmission routes. Although zoonotic exposure remains an important risk factor, transmission from a wide range of animal species contributes to human infection. Domestic ruminants, particularly cattle, sheep and goats, act as primary reservoirs, but wild animals and pets also play a role in the dissemination of the pathogen [3]. Inhalation of contaminated aerosols represents the primary route of human infection, whereas percutaneous transmission can occur through tick bites [1]. Environmental contamination, including soil, dust and water is also critical, given that *C. burnetii* is excreted in high quantities in birth products, particularly after abortions [16]. Additional routes of transmission include ingestion of contaminated or undercooked food of animal origin and, more rarely, direct human‐to‐human contagion. Sexual transmission has been suggested, as viable *C. burnetii* has been detected in spermatic fluid [17, 18].

It is currently assumed that QF infection induces long‐lasting immunity; however, a distinction must be made between humoral and cellular responses. Antibody titres (IgG) are known to decline within a few years after infection but are detectable up to 10 years after vaccination [19, 20]. In contrast, memory T‐cells remain detectable for considerably longer periods and likely contribute to long‐term immunity [21]. As routine diagnostics rely mainly on antibody testing, the interpretation of seroprevalence data remains challenging.

This study analysed prevalence of *C. burnetii*–specific antibodies among blood donors in Baden‐Wuerttemberg and Hesse, Germany. We investigated regional seroprevalence, relevant occupational and exposure risk factors, and age‐dependent antibody patterns.

## 2 Methods

### 2.1 Study Population

A serological survey in blood donors was conducted in Baden‐Wuerttemberg and Hesse in 2014. As part of the multicentre cross‐sectional study, a two‐stage cluster sampling was performed. In the first stage, in each of seven administrative districts—Darmstadt, Giessen, Kassel, Karlsruhe, Stuttgart, Tuebingen and Freiburg—30 donation events per district were randomly selected, yielding a total of 210 blood donation events. These donation events were further subdivided according to community type, yielding ten events each in predominantly urban, semi‐urban and rural communities, respectively. The classification of these community types was based on the municipal register of the Federal Statistical Office (data from the municipal register degree of urbanization by area, population and population density) [22].

**FIGURE 1:**
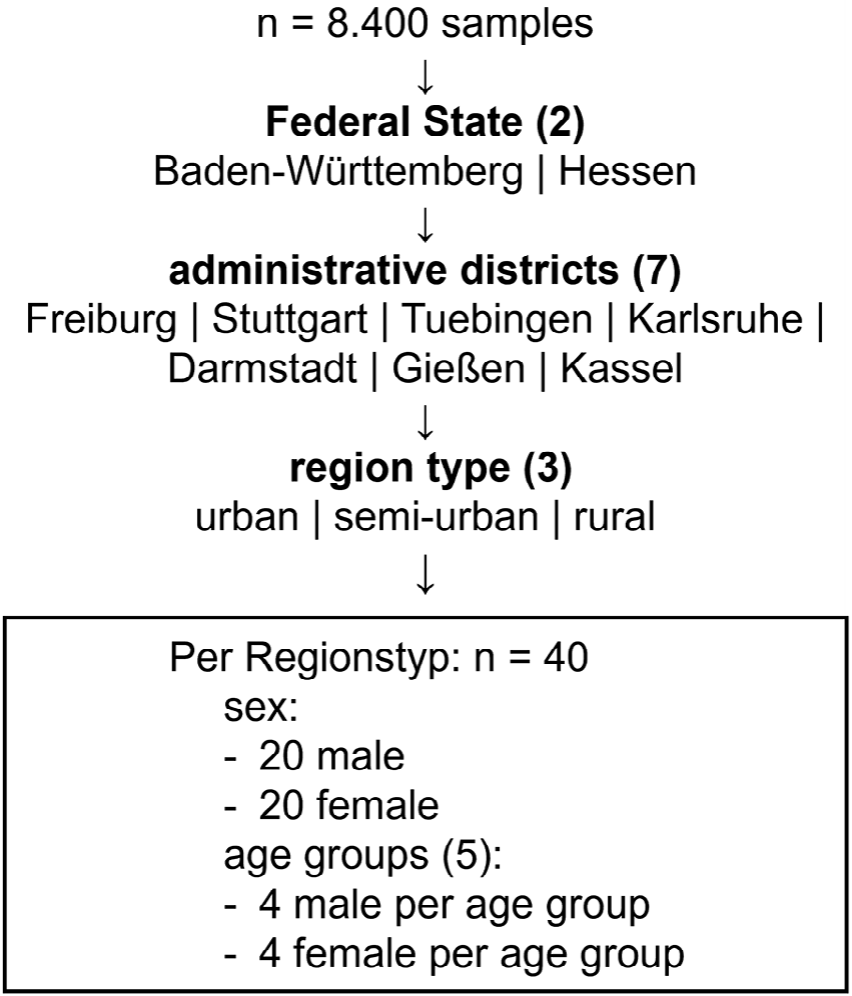
Overview of the stratified sampling design. The sample (N = 8,400) was stratified by federal state, administrative district, and region type (urban, semi‐urban, rural). Within each region type, equal numbers of participants were sampled across sex and age‐group strata (n = 40 per region type).

In the second stage, up to 40 donors were recruited at each donation event stratified by age group (*n* = 5) and sex (*n* = 2), means an equal sex distribution was targeted—20 participants of each sex. Five age groups were defined, with four participants from each group (Figure 1).

### 2.2 Questionnaire

Study participants were requested to fill in a standardized questionnaire, reporting demographical data (e.g., age, gender, place of residence and employment) and potential risk factors such as contact with animals, duration of outdoor stay during work and free time, health and habits (e.g., nutrition and smoking). Place of residence was recorded using the postal code and subsequently classified as rural, semi‐urban or predominantly urban, based on data from the Federal Statistical Office of Germany [22].

Five particular occupations (agriculture, veterinary, etc.) associated with close animal contact were selectable in the questionnaire. The participants were asked whether they had *ever* worked in one of these occupations in their lives, regardless of when and for how long.

In addition, participants indicated how often they had contact with animals or stayed in defined areas such as farmland, shed/field or forest in the last 3 years. For analysis, ‘several times per week’ and ‘several times per month’ were defined as ‘frequently’, whereas once a month or less was defined as ‘rarely’. Furthermore, participants were asked how often they had noticed tick bites in the last 3 years. Upon request, the participants were assisted by a trained interviewer. The full questionnaire is provided as Supporting Information.

### 2.3 Collection of Blood Samples

Blood samples were collected during the regular blood donation. No separate blood draw was necessary for the study participants. Blood samples were collected by standard venipuncture without the use of anticoagulants. After clotting, serum was obtained by centrifugation and subsequently aliquoted and stored frozen until analysis. Samples were distributed to the participating laboratories under appropriate conditions.

For the detection of *C. burnetii*, all serum samples were initially analysed at the same time using ELISA. Subsequently, indirect immunofluorescence assay (IFA) was performed (assay details are provided below). All samples were processed under comparable conditions to ensure consistency. In total, 5215 blood samples were collected.

### 2.4 Laboratory Investigations

A total number of 5218 blood donors in Baden‐Wuerttemberg and Hesse participated in the study. For three participants, no serum sample was obtained. Therefore, sera of 5215 blood donors were tested for anti‐*C. burnetii* antibodies by ELISA (Virion/Serion ELISA classic *C. burnetii*, Fa. Virion, Wuerzburg, sensitivity: IgG = 92.5%, IgM = 94.4%; specificity: IgG = >99%, IgM = 99.3%). Reactive sera were confirmed by semi‐quantitative indirect immunofluorescence assay (IFA, Focus Diagnostics, Cypress, CA, USA, sensitivity: IgG = 100%, IgM = 100%; specificity: IgG = 99%, IgM = 100%), the reference method for serological diagnosis of acute and chronic QF. All tests were performed according to the manufacturer's instructions. Negative and positive controls were included in each test run as fluorescence standard. IgM and IgG against Phases I and II antigens were analysed: detection of IgM antibodies indicates an acute infection, whereas IgG phase II antibodies indicate a previous infection. Persistently high IgG phase I titre indicate chronic QF [20, 23].

### 2.5 Case Definition

In this study, past QF cases were defined as having Phase II IgG antibody titre ≥64. A possible chronic course of QF cases was defined as having Phase I IgG antibody titre of ≥1024. Sera were also tested for Phases I and II IgM antibodies to detect acute QF, which was defined with a titre ≥64.

### 2.6 Inclusion and Exclusion Criteria

Eligible blood donors who provided a sample and gave voluntary consent were enrolled in the study. First‐time blood donors were excluded from participation. For specific analyses, participants were excluded when relevant data were missing. For all analyses, all participants who answered the corresponding questions in the questionnaire were included.

### 2.7 Statistical Analysis

Data were managed using Excel 2024 (Microsoft). For associations between serological test results and possible risk factors, relative risks (RR) were calculated. For these analyses, varying denominators were applied, reflecting the number of respondents with valid responses to each specific questionnaire item. Consequently, sample sizes differ between analyses. The age‐dependent diagrams were created using RStudio (Version 2024.4.2). A total of 5218 participants were recruited for the study. Blood samples were missing for 3 participants, and 44 provided insufficient or implausible questionnaire data and were therefore excluded. Consequently, data from 5171 participants were included in the statistical analysis.

## 3 Results

### 3.1 Study Population

In 2014, a total number of 5218 blood donors in Baden‐Wuerttemberg and Hesse participated in the study. For three participants, no serum sample was obtained. Therefore, sera of 5215 blood donors were serologically tested for anti‐*C. burnetii* antibodies. A total of 44 participants provided incomplete or incorrect information about their gender and/or age and were excluded from the analysis.

The age of the participants ranged from 18 to 70 years with a median of 49 years; 2652 were male (51.3%), and 2519 were female (48.7%). 1793 participants (34.7%) lived in rural areas, 1604 (31.0%) in semi‐urban areas and 1694 (32.8%) in predominantly urban areas (80 participants gave wrong information about their place of residence, e.g., postal code does not exist).

### 3.2 Seroprevalence of Anti‐*C. burnetii* Antibodies

In total, sera of *n* = 153 (2.96%) donors were tested positive for anti‐*C. burnetii* antibodies. Of these, *n* = 149 were tested positive for Phase II IgG antibodies, indicating past QF infection, whereas *n* = 4 were also tested positive for Phase II IgM antibodies, indicating acute QF illness (Table 1). High Phase I IgG antibody titres (≥1024) are an indicator for a possible chronic course of QF. Such a high titre was detected in four donors. None of these individuals reported a prior diagnosis of QF.

**TABLE 1:** Seroprevalence of anti‐*Coxiella burnetii* antibodies.

Q fever	*N*	%	IFA	Phase I ≥1024	Phase II ≥64
Negative	5018	97.04	**Positive IgG**	4	153
Positive	153	2.96	**Positive IgM**		4
Total	5171	100			

**FIGURE 2:**
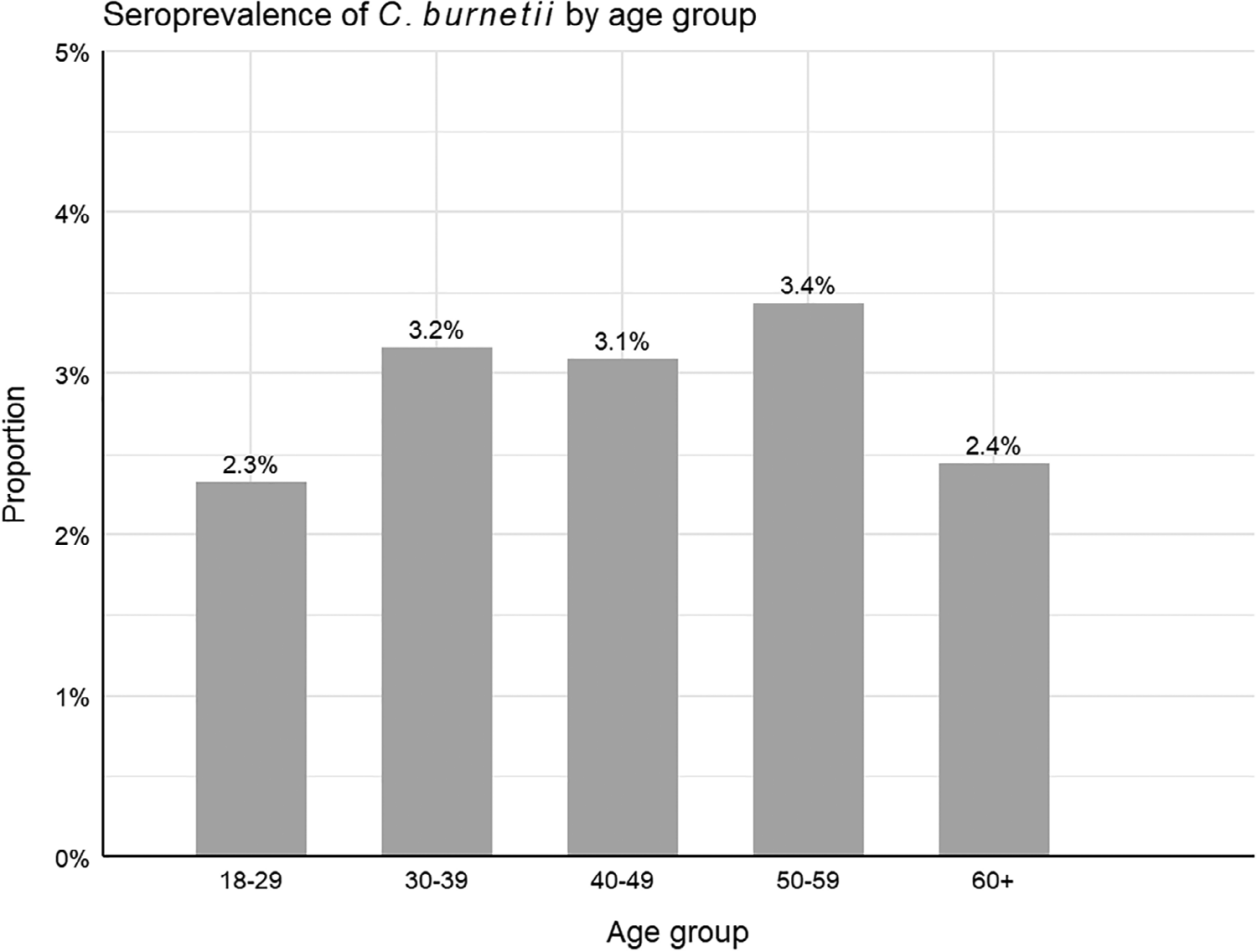
Seroprevalence of C. burnetii by age group.

To analyse how seroprevalence changes over age, age groups were defined, revealing the highest positive rate between 50 and 59 years (49/1426, 3.4%) and the lowest rate among the age group of 18–29 years (17/731, 2.3%). Overall, the variations of seropositivity across age groups were between 2.5% and 3.5% (Figure 2).

### 3.3 Findings of Questionnaire: Gender and Residence

The RR of being positive for *C. burnetii* was higher among participants from rural (RR = 1.76, 67/1793) and semi‐urban (RR = 1.40, 48/1604) areas compared with those from predominantly urban areas (reference group) (Table 2. Furthermore, male participants were more often seropositive (91/2652; 3.43%) compared to female participants (62/2519; 2.46%), corresponding to an RR of 1.39 (Table 2).

**TABLE 2:** Number of participants by place of residence and gender.

	% positive	RR
**Residence**		
Rural	3.74 (67/1793)	1.76
Semi‐urban	2.99 (48/1604)	1.40
Predominantly urban	2.13 (36/1694)	Reference group
Missing information on residence	2.50 (2/80)	
**Gender**		
Male	3.43 (91/2652)	1.39
Female	2.46 (62/2519)	Reference group

Abbreviation: RR, relative risk.

### 3.4 Findings on Questionnaire: Occupations Presenting a High Risk of Exposure

No participant was excluded due to missing residence or gender data. Table 3 summarizes the distribution of occupational exposures and antibody status among participants.

**TABLE 3:** Relative risks of *Coxiella burnetii* seropositivity for participants who had ever worked in an occupation with close animal contact in their lifetime.

	Yes % positive	No % positive	RR
**Occupation**			
Shepherd/Shearer	10.64 (5/47)	2.77 (134/4829)	3.83
Veterinary	5.17 (3/58)	2.78 (134/4815)	1.86
Agriculture/Farming	5.59 (48/859)	2.45 (101/4120)	2.28
Forester/Forestry worker	4.67 (19/407)	2.72 (122/4486)	1.72
Abattoir worker	9.40 (14/149)	2.66 (126/4730)	3.53
**Any of these jobs (high‐risk occupations)**	5.18 (56/1082)	2.43 (95/3907)	2.13

Abbreviation: RR, relative risk.

For individual occupations, the highest RR was observed for shepherds/shearers, with QF positivity being 3.83 (5/47 vs. 134/4829) times as high as that of those not working in this job. Participant who had ever worked as abattoir workers had an RR 3.53 (14/149 vs. 126/4730) times as high as people who never worked in this job, whereas individuals who had ever worked in agriculture/farming had an RR 2.28 (48/859 vs. 101/4120) times as high as those not working in this job. Veterinary work was associated with an RR 1.86 (3/58 vs. 134/4815) times as high as others, and former forestry workers with an RR 1.72 (19/407 vs. 122/4486) times as high as that of those not working in this job. All selectable occupations were summarized in a new variable named ‘high‐risk occupations’ (positive if at least one occupation was answered with ‘yes’, negative otherwise unless the blood donor did not answer any of the occupation questions). The analysis showed that QF positivity was 5.18% (56/1082) for people who had ever worked in a *high‐risk occupation*, whereas in the group without high‐risk occupations, the prevalence was only 2.43% (*n* = 95/3907).

In terms of environmental exposure (Table 4), living or spending time in farmland areas in the last 3 years was associated with an RR of 1.58 (76/2033 vs. 60/2538) compared to people who only rarely spend time on farmland, while staying in sheds or fields was linked to an RR of 2.61 (36/584 vs. 87/3684). In contrast, spending time in the last 3 years in forests showed a lower RR of only 0.87 (74/2717 vs. 66/2119) as high as staying rarely in forests. Participants who reported any of these risk areas had only a slightly elevated risk (RR = 1.09, 99/3279 vs. 39/1406).

**TABLE 4:** Relative risks of *Coxiella burnetii* seropositivity for staying in risk areas or contact to animals in the last 3 years and for other exposure factors.

	Frequently % positive	Rarely % positive	RR
**Risk areas**			
Farmland	3.74 (76/2033)	2.36 (60/2538)	1.58
Shed/Field	6.16 (36/584)	2.36 (87/3684)	2.61
Forest	2.72 (74/2717)	3.11 (66/2119)	0.87
**Any of these risk areas**	3.02 (99/3279)	2.77 (39/1406)	1.09
**Contact with**			
Sheep	6.35 (8/126)	2.62 (101/3860)	2.43
Goat	7.29 (7/96)	2.58 (99/3842)	2.83
Cattle	8.57 (27/315)	2.44 (92/3777)	3.52
**Any of these contacts**	7.38 (31/420)	2.40 (86/3578)	3.07
**Others**			
Consume raw milk	3.57 (20/560)	2.75 (103/3748)	1.30
Smoking	3.47 (31/893)	2.87 (122/4253)	1.21
Reported tick bites	3.12 (7/244)	2.94 (140/4756)	0.97

Abbreviation: RR, relative risk.

Regarding animal contact, the highest RR was observed for cattle contact (RR = 3.52, 27/315 vs. 92/3777), followed by goat contact (RR = 2.83, 7/96 vs. 99/3842) and sheep contact (RR = 2.43, 8/126 vs. 101/3860). Reporting any one of these contacts resulted in a risk 3.07 (31/420 vs. 86/3578) times as high as having no contacts with one of these animals (Table 4).

For other exposures, summarized in Table 4, raw milk consumption was associated with an RR 1.30 (20/560 vs. 103/3748) times as high for people who frequently consumed raw milk compared to people who rarely consumed raw mild, and smoking with an RR 1.21 (31/839 vs. 122/4253) times as high as that of non‐smokers. Reporting tick bites in the last 3 years, however, showed a slightly lower risk (RR = 0.97, 7/244 vs. 140/4756).

### 3.5 Findings on Questionnaire: Age and High‐Risk Occupation

**FIGURE 3:**
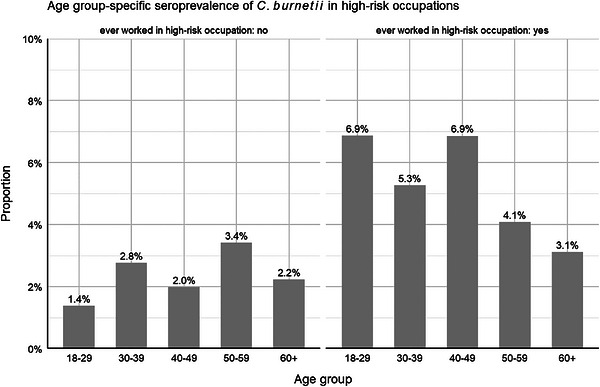
Age group‐specific seroprevalence of C. burnetii in high‐risk occupations.

As the newly created variable ‘high‐risk occupation’ showed an increased risk of positivity, we decided to further examine these two new groups by analysing how seropositivity was influenced by age (Figure 3). Although participant who never worked in a high‐risk occupation lack a clear age dependency, the people who had worked in a high‐risk occupation were more often positive below the age of 50 years.

## 4 Discussion

This study provides, for the first time, basic data on the seroprevalence of *C. burnetii* in a non‐risk population of blood donors in Baden‐Wuerttemberg and Hesse in Germany. The data indicate a seroprevalence of anti‐*C. burnetii* antibodies of approximately 3%, with place of residence and animal contact identified as important risk factors, alongside occupations involving close animal exposure. Additionally, it was observed that within high‐risk occupational groups, seroprevalence is higher among younger individuals compared to older age groups.

A unique feature of this study is that it does not only focus on classical risk groups such as abattoir workers, farmers or veterinarians but also includes blood donors from the general population. Another strength is the large sample size of over 5000 participants included in the analysis.

A comparable study from Australia, conducted on more than 2700 blood donors between 2014 and 2015, found a seroprevalence was 3.6%, which is similar to the result reported in the present study [24]. The seroprevalence in the general population aged 20 years and older in the United States is reported as 3.1% [25]. In Europe, a meta‐analysis on the epidemiology of QF in humans from 1982 to 2010 examined case studies from Bulgaria, France, Germany and the Netherlands [26]. In the Netherlands, seroprevalence ranged from 2.4% (general population) to 12.2% (blood donors) before and after the large outbreak from 2007 to 2010 [27, 28]. French studies reported seroprevalence values between 1.0% and 4.0% in blood donors [29, 30]. Only one German study was cited in the meta‐analysis, showing a prevalence of 22.0%, but in a risk group of farmers whose livestock experienced abortions [31].

QF is mostly asymptomatic, mild or self‐limiting [1, 32]. Symptoms are usually nonspecific, making diagnosis difficult. The gold standard for diagnosing *C. burnetii* infection is serological testing, which measures the humoral immune response by detecting antibodies. However, antibody levels decline over time, limiting the long‐term usefulness of serology. To address this, alternative assays have been developed, which measure *C. burnetii*‐specific T‐cell responses and thus evaluate the cellular immune response [21, 23]. Cellular immune responses, especially T‐cell‐mediated immunity, play a crucial role in controlling infection. Studies in mice models indicate that T‐cells play a crucial role in infection control, as antibodies alone are often inadequate to effectively eliminate the infection. Furthermore, memory T‐cell responses persist substantially longer than antibody levels, contributing to durable immunity [19, 21]. Therefore, the detection of T‐cells using a whole‐blood IFN‐γ release assay (IGRA) may also be a more suitable diagnostic tool for diagnosing long‐term effects such as chronic QF [33]. Chronic QF is a serious complication, which can result in death from endocarditis or vascular aneurysms [34]. This highlights the importance of accurate and early diagnosis, treatment and follow‐up care after a QF infection. Several studies have shown that QF is an underdiagnosed and underestimated illness [34, 35, 36]. The present study revealed a seroprevalence of 2.96%, which may still be an underestimate, as has been hinted by the lower seropositivity in older age groups. Interestingly, none of the positive individuals reported a prior diagnosis of QF, indicating that their infection had gone unnoticed. This underlines how underdiagnosed QF is and highlights the need for increased awareness of this serious disease.

As a zoonosis, human QF disease is usually associated with contact to animals, which was confirmed in this study by the fact that contact to animals in the last 3 years represented the highest risk of being *C. burnetii* positive. Animals associated with QF are mainly ruminant farm animals, which are naturally found in rural areas, suggesting that rural residents may be more frequently affected than urban residents. An Australian study also revealed a higher seropositivity in rural areas compared to urban areas and highest seropositivity for rural residence with regular contact to animals (like sheep, cattle or goat) [24]. The present study confirmed this assumption, showing more seropositive individuals living in rural areas compared to urban areas.

According to the literature, the type of occupation is an important risk factor for acquiring a QF illness. Recent studies have focused on groups with frequent animal contact, such as animal husbandry and abattoir workers, who show increased infection risk [37]. High prevalence in veterinarians, agricultural workers and forestry workers further confirmed an occupational risk [6, 31, 38], and these observations were also confirmed in our study. However, we did not focus only on high‐risk occupations but also examined the age‐dependent course of prevalence in these high‐risk occupations. Among participants who ever worked in a *high‐risk occupation*, seroprevalence appeared to form a midlife plateau and then declined in older age groups. This pattern could be explained by the combination of the following (hypothetical) assumptions: Antibody titres fall below detection thresholds within a few years (Teunis et al. estimated the half‐life of *C. burnetii*–specific IgG to be ∼318 days) [19, 20, 21]. Furthermore, exposure is likely more intense during active working years in high‐risk occupations such as shepherding, veterinary practice or abattoir work. With increasing age, individuals may change roles, reduce occupational exposure or retire, potentially leading to fewer opportunities for infections and for immune boosting and consequently lower antibody prevalence in older groups. However, this explanation remains hypothetical, as these factors were not directly assessed in our study.

Evidence from occupational cohorts supports this interpretation. In a 3‐year follow‐up study, Wielders et al. demonstrated that veterinarians maintained significantly higher IgG phase I titres compared to patients with a single acute infection, suggesting that repeated exposure can sustain antibody levels over time [39]. In contrast, older individuals who no longer work in high‐risk occupations are less likely to experience infection or boosting, accelerating the visible decline of seropositivity. In addition, age‐related changes in the immune system, including immunosenescence, may influence the magnitude and persistence of antibody responses in older individuals and thereby contribute to the observed decline in seroprevalence [40].

Overall, the combination of antibody waning and changing exposure over the life course provides a coherent explanation for the midlife peak and the decline at older ages. As routine diagnostics rely mainly on serology, prior infections in older groups are likely underestimated. Future studies should, therefore, complement serology with T‐cell–based assays (e.g., IGRA and ELISPOT) to better capture long‐term immunity across age groups [19, 21].

In summary, the seroprevalence of QF in the present study population is 2.96%, consistent with studies from other countries, such as Australia, the United States and other European countries. As expected, *C. burnetii* seropositivity is more common in people who have ever worked in agricultural, abattoir and forestry occupations. The observation that people living in rural areas are more often affected than people living in urban areas was also expected. The most striking finding was that none of the seropositive participants had ever been knowingly diagnosed with QF, illustrating how much this disease remains underdiagnosed.

## 5 Limitations

This study has several limitations. The study population consisted only of blood donors, who may not represent the general population. The cross‐sectional design and availability of only one serum sample prevent conclusions on time of infection, antibody waning or immune dynamics over time. Questionnaire items were partly inconsistent, with varying time frames, which limits comparability: Participants were asked about stays in risk areas and contact with animals during the past 3 years; for raw‐milk consumption there was no time limit, and tick bites were also recorded for the past 3 years. Occupations were recorded only as *ever employed*, without information on when participants worked in this occupation and for how long. This temporal ambiguity is particularly important for older donors, who may have worked in high‐risk occupations decades ago but subsequently lost detectable antibodies. Longitudinal studies including both serology and cellular immunity markers are needed to address these gaps.

## Author Contributions

*Conceptualization:* Volkhard A. J. Kempf, Mirko Faber, Kai Hourfar and Silke F. Fischer. *Data curation:* Larissa Dangel and Martin Eichner. *Formal analysis:* Larissa Dangel and Martin Eichner. *Funding acquisition:* Volkhard A. J. Kempf and Silke F. Fischer. *Investigation:* Daniela Hummel and Kai Hourfar. *Methodology:* Volkhard A. J. Kempf, Mirko Faber, Kai Hourfar and Silke F. Fischer. *Project administration:* Larissa Dangel. *Resources:* Volkhard A. J. Kempf, Mirko Faber and Kai Hourfar. *Supervision:* Silke F. Fischer. *Visualization:* Larissa Dangel. *Writing – original draft:* Larissa Dangel. *Writing – review and editing:* Larissa Dangel, Volkhard A. J. Kempf, Mirko Faber, Brockmann Stefan and Martin Eichner.

## Funding

The project was partially funded by the Robert Koch‐Institute, Berlin, Germany (‘Netzwerk Zoonosen’, FKZ: 1369‐432 and Coxiella Consiliary Laboratory, FKZ 1369‐358).

## Disclosure

The first author is a fellow of the ECDC Fellowship Programme, supported financially by the European Centre for Disease Prevention and Control (ECDC). The views and opinions expressed herein do not state or reflect those of ECDC. ECDC is not responsible for the data and information ECDC FELLOWSHIP PROGRAMME 2 ECDC NORMAL collation and analysis and cannot be held liable for conclusions or opinions drawn.

## Ethics Statement

This study was approved by the ethical review committee of the university hospital Frankfurt (Votum ‘190/14’ August 2014).

## Conflicts of Interest

The authors declare no conflicts of interest.

## Supporting information




**Supporting File 1**: puh270284‐sup‐0001‐SuppMat.pdf

## Data Availability

The data that support the findings of this study are available on request from the corresponding author. The data are not publicly available due to privacy or ethical restrictions.

## References

[1] M. Maurin and D. Raoult , “Q Fever,” Clinical Microbiology Reviews 12 (1999): 518–553, 10.1128/CMR.12.4.518. 10515901 PMC88923

[2] E. H. Derrick , ““Q” FEVER, A New Fever Entity: Clinical Features, Diagnosis And Laboratory Investigation,” Medical Journal of Australia 2, no. 8 (1937): 281–299, 10.5694/j.1326-5377.1937.tb43743.x. 6622891

[3] C. E. Delsing , B. J. Kullberg , and C. P. Bleeker‐Rovers , “Q Fever in the Netherlands From 2007 to 2010,” Netherlands Journal of Medicine 68, no. 12 (2010): 382–387. 21209463

[4] K. Boden , S. Brasche , E. Straube , and W. Bischof , “Specific Risk Factors for Contracting Q Fever: Lessons From the Outbreak Jena,” International Journal of Hygiene and Environmental Health 217, no. 1 (2014): 110–115, 10.1016/j.ijheh.2013.04.004. 23707055

[5] A. Hilbert , P. Reith , S. O. Brockmann , et al., “Epidemiologische Untersuchungen zu zwei Q‐Fieber‐Ausbrüchen in einer Gemeinde Baden‐Württembergs in den Jahren 2008 und 2009,” Berliner und Munchener Tierarztliche Wochenschrift 124 (2011): 295–302. 21848037

[6] A. Jurke , N. Bannert , K. Brehm , et al., “Serological Survey of *Bartonella* spp., *Borrelia burgdorferi*, *Brucella* spp., *Coxiella burnetii*, *Francisella tularensis*, *Leptospira* spp., *Echinococcus*, Hanta‐, TBE‐ and XMR‐Virus Infection in Employees of Two Forestry Enterprises in North Rhine‐Westphalia, Germany, 2011–2013,” International Journal of Medical Microbiology: IJMM 305 (2015): 652–662, 10.1016/j.ijmm.2015.08.015. 26422407

[7] S. M. Woldeyohannes , C. F. Gilks , P. Baker , N. R. Perkins , and S. A. Reid , “Seroprevlance of *Coxiella burnetii* Among Abattoir and Slaughterhouse Workers: A Meta‐Analysis,” One Health (Amsterdam, Netherlands) 6 (2018): 23–28, 10.1016/j.onehlt.2018.09.002. 30302365 PMC6175780

[8] B. Schimmer , N. Schotten , E. van Engelen , J. L. A. Hautvast , P. M. Schneeberger , and Y. T. H. P. van Duijnhoven , “ *Coxiella burnetii* Seroprevalence and Risk for Humans on Dairy Cattle Farms, the Netherlands, 2010–2011,” Emerging Infectious Diseases 20 (2014): 417–425, 10.3201/eid2003.131111. 24572637 PMC3944848

[9] J. S. Agerholm , T. K. Jensen , J. F. Agger , M. Y. Engelsma , and H. I. J. Roest , “Presence of *Coxiella burnetii* DNA in Inflamed Bovine Cardiac Valves,” BMC Veterinary Research 13 (2017): 69, 10.1186/s12917-017-0988-5. 28274243 PMC5343293

[10] T. Chmielewski and S. Tylewska‐Wierzbanowska , “Q Fever at the Turn of the Century,” Polish Journal of Microbiology 61 (2012): 81–93, 10.33073/pjm-2012-011. 23163207

[11] C. C. H. Wielders , J. A. F. van Loenhout , G. Morroy , et al., “Long‐Term Serological Follow‐Up of Acute Q‐Fever Patients After a Large Epidemic,” PlOS one 10 (2015): e0131848, 10.1371/journal.pone.0131848. 26161658 PMC4498618

[12] C. C. H. Wielders , G. Morroy , P. C. Wever , R. A. Coutinho , P. M. Schneeberger , and W. van der Hoek , “Strategies for Early Detection of Chronic Q‐Fever: A Systematic Review,” European Journal of Clinical Investigation 43 (2013): 616–639, 10.1111/eci.12073. 23550525

[13] L. M. Kampschreur , C. E. Delsing , R. H. H. Groenwold , et al., “Chronic Q Fever in the Netherlands 5 Years After the Start of the Q Fever Epidemic: Results From the Dutch Chronic Q Fever Database,” Journal of Clinical Microbiology 52 (2014): 1637–1643, 10.1128/JCM.03221-13. 24599987 PMC3993626

[14] S. E. van Roeden , P. C. Wever , L. M. Kampschreur , et al., “Chronic Q Fever‐Related Complications and Mortality: Data From a Nationwide Cohort,” Clinical Microbiology and Infection: The Official Publication of the European Society of Clinical Microbiology and Infectious Diseases 25 (2019): 1390–1398, 10.1016/j.cmi.2018.11.023. 30543852

[15] P. M. Schneeberger , M. H. A. Hermans , E. J. van Hannen , J. J. A. Schellekens , A. C. A. P. Leenders , and P. C. Wever , “Real‐Time PCR With Serum Samples is Indispensable for Early Diagnosis of Acute Q Fever,” Clinical and Vaccine Immunology: CVI 17, no. 2 (2010): 286–290, 10.1128/CVI.00454-09. 20032219 PMC2815520

[16] A. Joulié , E. Rousset , P. Gasqui , et al., “ *Coxiella burnetii* Circulation in a Naturally Infected Flock of Sheep: Individual Follow‐Up of Antibodies in Serum and Milk,” Applied and Environmental Microbiology 83 (2017): e00222–17, 10.1128/AEM.00222-17. PMC5479003 28455328

[17] M. H. Miceli , A. K. Veryser , A. D. Anderson , D. Hofinger , S. A. Lee , and C. Tancik , “A Case of Person‐to‐Person Transmission of Q Fever From an Active Duty Serviceman to His Spouse,” Vector Borne and Zoonotic Diseases (Larchmont, New York) 10 (2010): 539–541, 10.1089/vbz.2009.0101. 20020811

[18] A. Milazzo , R. Hall , P. A. Storm , R. J. Harris , W. Winslow , and B. P. Marmion , “Sexually Transmitted Q Fever,” Clinical Infectious Diseases: An Official Publication of the Infectious Diseases Society of America 33 (2001): 399–402, 10.1086/321878. 11438911

[19] G. J. Kersh , K. A. Fitzpatrick , J. S. Self , B. J. Biggerstaff , and R. F. Massung , “Long‐Term Immune Responses to *Coxiella burnetii* After Vaccination,” Clinical and Vaccine Immunology: CVI 20 (2012): 129–133, 10.1128/CVI.00613-12. 23192629 PMC3571276

[20] P. F. M. Teunis , B. Schimmer , D. W. Notermans , et al., “Time‐Course of Antibody Responses Against *Coxiella burnetii* Following Acute Q Fever,” Epidemiology and Infection 141 (2013): 62–73, 10.1017/S0950268812000404. 22475210 PMC9152070

[21] A. Scholzen , G. Richard , L. Moise , et al., “ *Coxiella burnetii* Epitope‐Specific T‐Cell Responses in Patients With Chronic Q Fever,” Infection and Immunity 87 (2019): e00213–e00219, 10.1128/IAI.00213-19. 31331958 PMC6759312

[22] Statistischen Bundesamt , Daten aus dem Gemeindeverzeichnis Grad der Verstädterung nach Fläche, Bevölkerung und Bevölkerungsdichte , EU‐Grad der Verstädterung (Statistischen Bundesamt, 2023).

[23] A. E. Sluder , S. Raju Paul , L. Moise , et al., “Evaluation of a Human T Cell‐Targeted Multi‐Epitope Vaccine for Q Fever in Animal Models of *Coxiella burnetii* Immunity,” Frontiers in Immunology 13 (2022): 901372, 10.3389/fimmu.2022.901372. 35651616 PMC9149306

[24] H. F. Gidding , H. M. Faddy , D. N. Durrheim , et al., “Seroprevalence of Q Fever Among Metropolitan and Non‐Metropolitan Blood Donors in New South Wales and Queensland, 2014–2015,” Medical Journal of Australia 210 (2019): 309–315, 10.5694/mja2.13004. 30848517

[25] A. D. Anderson , D. Kruszon‐Moran , A. D. Loftis , et al., “Seroprevalence of Q Fever in the United States, 2003–2004,” American Journal of Tropical Medicine and Hygiene 81 (2009): 691–694, 10.4269/ajtmh.2009.09-0168. 19815888

[26] M. Georgiev , A. Afonso , H. Neubauer , et al., “Q Fever in Humans and Farm Animals in Four European Countries, 1982 to 2010,” Euro Surveillance: Bulletin Europeen sur les Maladies Transmissibles = European Communicable Disease Bulletin 18 (2013): 20407. 23449232

[27] B. Schimmer , D. W. Notermans , M. G. Harms , et al., “Low Seroprevalence of Q Fever in The Netherlands Prior to a Series of Large Outbreaks,” Epidemiology and Infection 140 (2012): 27–35, 10.1017/S0950268811000136. 21324217

[28] B. M. Hogema , E. Slot , M. Molier , et al., “ *Coxiella burnetii* Infection Among Blood Donors During the 2009 Q‐Fever Outbreak in the Netherlands,” Transfusion 52 (2012): 144–150, 10.1111/j.1537-2995.2011.03250.x. 21756265

[29] H. Tissot‐Dupont , V. Vaillant , S. Rey , and D. Raoult , “Role of Sex, Age, Previous Valve Lesion, and Pregnancy in the Clinical Expression and Outcome of Q Fever After a Large Outbreak,” Clinical Infectious Diseases: An Official Publication of the Infectious Diseases Society of America 44 (2007): 232–237, 10.1086/510389. 17173223

[30] S. Esmaeili , S. R. Naddaf , B. Pourhossein , et al., “Seroprevalence of Brucellosis, Leptospirosis, and Q Fever Among Butchers and Slaughterhouse Workers in South‐Eastern Iran,” PlOS One 11 (2016): e0144953, 10.1371/journal.pone.0144953. 26731333 PMC4701462

[31] R. Sting , J. Kopp , J. Mandl , et al., “ *Coxiella burnetii*‐Infektionen in Milchviehbetrieben unter besonderer Berücksichtigung von Infektionen bei Menschen,” Berliner und Munchener Tierarztliche Wochenschrift 115 (2002): 360–365. 12357673

[32] D. Raoult , T. Marrie , and J. Mege , “Natural History and Pathophysiology of Q Fever,” Lancet Infectious Diseases 5 (2005): 219–226, 10.1016/S1473-3099(05)70052-9. 15792739

[33] G. J. M. Limonard , S. F. Thijsen , A. W. Bossink , A. Asscheman , and J. J. M. Bouwman , “Developing a New Clinical Tool for Diagnosing Chronic Q Fever: The Coxiella ELISPOT,” FEMS Immunology and Medical Microbiology 64 (2012): 57–60, 10.1111/j.1574-695X.2011.00890.x. 22067057

[34] F. S. Dahlgren , D. L. Haberling , and J. H. McQuiston , “Q Fever is Underestimated in the United States: A Comparison of Fatal Q Fever Cases From Two National Reporting Systems,” American Journal of Tropical Medicine and Hygiene 92 (2015): 244–246, 10.4269/ajtmh.14-0502. 25404074 PMC4347323

[35] E. E. Sickbert‐Bennett , D. J. Weber , C. Poole , P. D. M. MacDonald , and J.‐M. Maillard , “Completeness of Communicable Disease Reporting, North Carolina, USA, 1995–1997 and 2000–2006,” Emerging Infectious Diseases 17 (2011): 23–29, 10.3201/eid1701.100660. 21192850 PMC3204630

[36] H. W. Kaufman , Z. Chen , J. Radcliff , H. J. Batterman , and J. Leake , “Q Fever: An Under‐Reported Reportable Communicable Disease,” Epidemiology and Infection 146 (2018): 1240–1244, 10.1017/S0950268818001395. 29941056 PMC9134298

[37] E. Mostafavi , L. Molaeipoor , S. Esmaeili , et al., “Seroprevalence of Q Fever Among High‐Risk Occupations in the Ilam Province, the West of Iran,” PlOS One 14 (2019): e0211781, 10.1371/journal.pone.0211781. 30779802 PMC6380538

[38] E. Sellens , K. L. Bosward , J. M. Norris , et al., “ *Coxiella burnetii* Seroprevalence in Unvaccinated Veterinary Workers in Australia: Evidence to Support Q Fever Vaccination,” Zoonoses and Public Health 67 (2020): 79–88, 10.1111/zph.12658. 31677254

[39] C. C. H. Wielders , A. W. Boerman , B. Schimmer , et al., “Persistent High IgG Phase I Antibody Levels Against *Coxiella burnetii* Among Veterinarians Compared to Patients Previously Diagnosed With Acute Q Fever After Three Years of Follow‐Up,” PlOS One 10 (2015): e0116937, 10.1371/journal.pone.0116937. 25602602 PMC4300228

[40] T. M. Doherty , B. Weinberger , A. Didierlaurent , and P.‐H. Lambert , “Age‐Related Changes in the Immune System and Challenges for the Development of Age‐Specific Vaccines,” Annals of Medicine 57 (2025): 2477300, 10.1080/07853890.2025.2477300. 40110678 PMC11926906

