# Supplementary material for: Coxiella burnetii Infection Among Blood Donors From Baden‐Wuerttemberg Province and Hesse Province, Germany: A Multicentre Cross‐Sectional Serological Study
Source: Public Health Chall. 2026 Jun 16;5(2):e70284. doi: 10.1002/puh2.70284 (PMC13270405; doi:10.1002/puh2.70284)
Supplement: Supplementary file 1 — Supporting File 1: puh270284‐sup‐0001‐SuppMat.pdf [file PUH2-5-e70284-s001.pdf]

Blood Donor Study Questionnaire

(Please affix barcode here)

Personal Information

**1. How old are you?**<br>_________________________
**2. What is your sex?**<br>☐ Male ☐ Female

3. In the past 3 years, how often have you spent time (privately or professionally) in the following environments?

	Several times/week	Several times/month	Less than once/month	Less than once/year	Don't know
Agricultural land	☐	☐	☐	☐	☐
Barn/pasture (sheep/goats/cattle)	☐	☐	☐	☐	☐
Forest	☐	☐	☐	☐	☐
Inland water bodies (stream, river, lake)	☐	☐	☐	☐	☐

4. In the past 3 years, how often have you had direct contact with livestock or pets?

	Several times/week	Several times/month	Less than once/month	Less than once/year	Don't know
Sheep	☐	☐	☐	☐	☐
Goat	☐	☐	☐	☐	☐
Cattle	☐	☐	☐	☐	☐
Pig	☐	☐	☐	☐	☐
Horse	☐	☐	☐	☐	☐
Cat	☐	☐	☐	☐	☐
Dog	☐	☐	☐	☐	☐
Rat	☐	☐	☐	☐	☐
Mouse	☐	☐	☐	☐	☐
Hare/Rabbit	☐	☐	☐	☐	☐

5. Have you ever worked, or are you currently working, in the following fields?

	Yes	No	Don't know
Shepherd / Shearer	☐	☐	☐
Veterinary medicine	☐	☐	☐
Agriculture	☐	☐	☐
Hunter	☐	☐	☐
Forester / forestry worker	☐	☐	☐
Slaughterhouse	☐	☐	☐
Wastewater	☐	☐	☐

6. Have you spent extended periods abroad (>4 weeks)?

☐ Yes ☐ No ☐ Don't know

If yes:

1. From: ______ to ______ Country: __________
2. From: ______ to ______ Country: __________
3. From: ______ to ______ Country: __________

---

7. Have you ever been diagnosed with any of the following diseases?

	Yes (year)	No	Don't know
Bartonellosis	☐ (____)	☐	☐
Lyme disease	☐ (____)	☐	☐
Tick-borne encephalitis (TBE)	☐ (____)	☐	☐
Hantavirus infection	☐ (____)	☐	☐
Leptospirosis	☐ (____)	☐	☐
Q fever	☐ (____)	☐	☐

8. Have you been vaccinated against the following diseases?

	Yes (year)	No	Don't know
TBE	☐ (____)	☐	☐
Smallpox	☐ (____)	☐	☐
Yellow fever	☐ (____)	☐	☐
Rabies	☐ (____)	☐	☐

If rabies vaccination:
☐ Occupational ☐ Travel ☐ Animal bite ☐ Other

If animal bite:
Year: ______ Country: ______ Animal: ______
Treated animal bite medically? ☐ Yes ☐ No

---

9. How often do you consume:

	Several times/week	Several times/month	Less than once/month	Less than once/year	Don't know
Raw milk	☐	☐	☐	☐	☐
Wild rabbit/hare	☐	☐	☐	☐	☐

---

10. Tick bites (last 3 years):
☐ None ☐ 1–5 ☐ 5–50 ☐ >50 ☐ Don't know

---

11. Mosquito bites (season):
☐ Several/week ☐ Several/month ☐ <1/month ☐ <1/year ☐ Don't know

---

12. Water-related sports?
☐ Yes ☐ No ☐ Don't know

---

13. Gardening frequency:

☐ Several/week ☐ Several/month ☐ <1/month ☐ <1/year ☐ Don’t know

14. Rodents (home/work):

☐ Several/week ☐ Several/month ☐ <1/month ☐ <1/year ☐ Don’t know

15. Smoking status:

☐ Daily: _____ cigarettes/day	☐ Occasionally	☐ Former smoker	☐ Never

16. Asthma:

☐ Yes ☐ No ☐ Don’t know
